# Resistance Training in Women Diagnosed with Breast Cancer: A Pilot Single Arm Pre–Post Intervention

**DOI:** 10.3390/cancers16162829

**Published:** 2024-08-12

**Authors:** Madison F. Vani, Maryam Marashi, Erin K. O’Loughlin, Jenna Smith-Turchyn, Catherine M. Sabiston

**Affiliations:** 1 Faculty of Kinesiology and Physical Education, University of Toronto, Toronto, ON M5S 2W6, Canada; madison.vani@mail.utoronto.ca (M.F.V.); maryam.marashi@mail.utoronto.ca (M.M.); erin.oloughlin@utoronto.ca (E.K.O.); 2 Centre de Recherche du Centre Hospitalier de L’université de Montréal (CRCHUM), Montréal, QC H2X 0A9, Canada; 3 School of Rehabilitation Sciences, McMaster University, Hamilton, ON L8S 1C7, Canada; smithjf@mcmaster.ca

**Keywords:** breast cancer, exercise, feasibility, peer-based intervention, resistance training, social support, virtual

## Abstract

**Simple Summary:**

Resistance training is important for women’s health and well-being, yet there are uncertainties whether resistance training can be delivered remotely to women diagnosed with breast cancer. This study explored the feasibility of a remotely delivered resistance training program. Also, physical activity studies rarely include peer support that may help amplify the effects of resistance training on key feasibility outcomes such as adherence and retention. This study aimed to explore the impact of qualified exercise professional and peer support in delivering a remote resistance training program. The findings suggest that resistance training can be delivered remotely, with support from qualified exercise professionals more beneficial than peer support. Further accountability needs to be offered to women to engage in resistance training autonomously. Based on the findings, resistance training offers favorable health and well-being outcomes. A larger trial is feasible and needed to inform cancer care.

**Abstract:**

**Background:** Resistance training (RT) yields physical and psychological benefits for women living with and beyond breast cancer (WBC). This study examined the feasibility of a virtually delivered 8-week socially supportive RT intervention among WBC and assessed changes in physical activity and body image. **Methods:** A pilot single-arm 8-week pre–post intervention study design was implemented. Forty-one WBC were matched as exercise partners and asked to complete two RT sessions per week—one with a qualified exercise professional (QEP) and one with their peer. Data were collected at baseline (T1), post-intervention (T2), and 20 weeks post-baseline (T3). **Results:** The enrolment rate was 42%, the attendance rate for the QEP sessions was 63.8% and 40.0% for the peer sessions, and the retention rate was 87%. No adverse events were reported. Total weekly minutes of RT significantly (*p* < 0.05) increased by 42 minutes/week during the intervention and significantly decreased by 25 min/week at follow-up. Upper and lower body muscle strength increased (*p* < 0.01) during the intervention. Increased RT was associated with favorable activity self-perceptions. **Conclusions:** This pilot intervention study was feasible, safe, and demonstrated preliminary evidence for increasing RT time and strength among WBC. Virtually delivered socially supportive RT interventions can improve access for WBC.

## 1. Introduction

The diagnosis and treatment of breast cancer is associated with several adverse physical and psychological side effects (e.g., pain, muscle mass reductions, fatigue, lymphedema, depressive symptoms, body image concerns) that can have long-term consequences on women living with and beyond breast cancer (WBC) [1,2]. Resistance training (RT, i.e., working against force to build strength, muscular endurance, and muscle mass) is a safe form of exercise that has been shown to be useful in managing acute and lasting effects of breast cancer [3,4,5]. Specifically, RT has been related to reductions in fatigue and pain, and improvements in quality of life, body image, muscular strength, and physical functioning among WBC [3,5]. Finally, RT does not increase the risk of lymphedema for WBC [3]. As a result of this evidence, cancer-specific guidelines recommend that WBC engage in RT at least two times per week [3,6].

While existing RT interventions have produced promising findings for individuals diagnosed with cancer, most RT protocols are delivered in-person and vary in the way social support is integrated [4]. Specifically, in-person interventions can reduce engagement and adherence to interventions due to limitations in accessibility (e.g., transportation, cost, scheduling) and comfort [7,8,9]. Providing virtually delivered home-based RT offers a safe alternative to in-person formats and may improve accessibility and retention [9,10,11,12]. Home-based and virtual exercise interventions have increased exercise-related self-efficacy and have improved muscle strength and physical activity behavior for WBC [11,13,14], yet all but one [14] delivered combined aerobic and RT interventions. There are limited RT-only interventions designed for WBC that are delivered virtually using a web-based platform [12]. It is important to independently evaluate the feasibility of virtually delivered RT interventions since RT requires specific skills and safety considerations [3,12]. Therefore, examining WBC’s adherence to a virtually delivered RT intervention and the effects of such an intervention on physical activity behavior, muscle strength, and RT self-efficacy would be valuable and is the main focus of the current study.

Additionally, WBC commonly report low social support as a barrier limiting their physical activity participation [7,8]. To address this barrier, physical activity interventions aimed at those diagnosed with cancer often provide opportunities for social interactions [15,16]. Indeed, socially supportive physical activity interventions improve adherence and increase physical activity behavior among WBC [15,17], facilitating social support through various sources (e.g., professionals, family, peers) offering information, encouragement, and shared experiences [15,17,18]. A qualified exercise professional (QEP, e.g., registered kinesiologists and physiotherapists) can uniquely offer WBC tailored feedback and encouragement in a supervised setting. QEP support can be particularly beneficial for WBC engaging in RT, given their difficulties accessing support from trained individuals who have knowledge about exercise and cancer [8]. Research among WBC shows that QEP-supervised RT leads to greater muscle strength when compared to unsupervised RT [19]. Additional benefits of engaging in supervised RT interventions for WBC have included increased physical activity behavior and higher exercise-related self-efficacy [14,20]. Of note, while some studies report participant satisfaction with the program (e.g., 14), WBC’s perceptions of the QEP are not described. As such, it may be important to assess the therapeutic alliance [21] between the QEP and WBC in RT-focused programs.

In addition to a QEP, peers also diagnosed with breast cancer may be uniquely equipped to provide each other with social support, since they both understand what it is like to experience breast cancer [17]. For example, Carr and colleagues [22] demonstrated that peer dyads may be particularly valuable for physical activity when compared to other dyad types (e.g., family, caregiver). WBC may especially benefit from engaging in peer support physical activity interventions, as many report challenges with finding a peer exercise partner [7,8]. Among WBC, remotely delivered peer support interventions had high retention rates and led to increased physical activity self-efficacy and physical activity behavior [17,23,24,25]. However, existing peer support interventions mainly involve trained peer mentors (i.e., one peer acts as the mentor for another peer) and aerobic physical activity only [17]. Peers who are matched together but not trained to be mentors may remove the uneven power dynamics that can emerge through peer mentor relationships and improve the mutual bond between peers [17,26]. Understanding peer support relationships among WBC engaging in RT is needed to amplify the outcomes of these health behavior interventions. In the current study, we tested whether combining peer-to-peer support and QEP support in RT interventions with WBC can enhance improvements in adherence, physical activity behavior, strength, and exercise-related self-efficacy. Given the importance of RT in improving body image and physical self-perceptions among WBC following cancer treatment [27,28,29,30,31], the current study also explored physical self-perceptions [31] as one of the integral outcomes of the intervention. 

The primary purpose of the current theoretically informed [32,33,34] single-arm pre–post pilot intervention study was to examine the feasibility of a virtually delivered and socially supportive RT intervention for WBC. Additionally, this pilot study assessed changes in RT, moderate-to-vigorous physical activity (MVPA), strength, RT self-efficacy, and physical self-perceptions. Feasibility was examined through (1a) enrolment rate, (1b) WBC’s adherence to the intervention, (1c) retention rate post-intervention and follow-up (20 weeks post-baseline), (1d) number of adverse events reported, (1e) participants’ ratings of their peer-match post-intervention, (1f) participant’s communication frequency and mode of communication with their peer match post-intervention and follow-up, and (1g) participants’ ratings of the therapeutic alliance with the QEP post-intervention. Given the exploratory nature of the main aims (1a-g), hypotheses were not generated. The secondary aims included to test changes in (2a) RT from baseline to post-intervention and follow-up, (2b) MVPA from baseline to post-intervention and follow-up, (2c) lower and upper-body strength from baseline to post-intervention, and (2d) RT self-efficacy from baseline to post-intervention and follow-up. We hypothesized that WBC would (2a) increase their RT, (2b) increase their MVPA, (2c) demonstrate improved strength, and (2d) increase their RT self-efficacy. The tertiary aims included examining (3a) changes in activity, appearance, body fat, coordination, flexibility, strength, and general physical self-perceptions from baseline to post-intervention and follow-up, and (3b) the association between changes in RT behavior, physical self-perceptions, RT self-efficacy, MVPA, and strength. We hypothesized that WBC would (3a) increase all physical self-perceptions, and (3b) RT change would be associated with increases in all physical self-perceptions, RT efficacy, MVPA, and strength.

## 2. Materials and Methods

### 2.1. Study Design and Participants

The current study was an optional 8-week RT protocol extension of the 2021–22 two-arm randomized controlled trial (RCT; Connecting Breast Cancer Survivors for Exercise (C4E)). The original study adhered to the CONSORT guidelines [35] and SPIRIT recommendations [36] for the reporting of clinical trials, and further details are published in the study protocol [37]. Briefly, the C4E trial evaluated whether WBC dyads who received 10 weekly sessions of virtually delivered QEP support had improved outcomes (MVPA volume, social support, quality of life) compared to WBC dyads who did not receive QEP support. The C4E study is registered on ClinicalTrials.gov (study identifier: NCT04771975, protocol Version Number: 2, date: 22 July 2021). 

This study extension was a single-arm pre–post intervention study design informed by participant feedback during the original RCT. Specifically, many participants described a need for RT yet reported significant barriers, such as a lack of resources, inadequate knowledge, and insufficient support to pursue such training independently. The current study was designed to address these challenges. As such, the current study eligibility and exclusion criteria were dependent on the original C4E RCT, and there was no new recruitment. Participants were eligible if they were (i) English-speaking females diagnosed with breast cancer; (ii) diagnosed with primary stage 0–IV breast cancer, at any stage of treatment; (iii) living in Canada; (iv) aged 18 years or older; (v) medically cleared for exercise; (vi) able to connect to the internet using any device (e.g., computer, tablet or smartphone; preferably with a webcam); and (vii) currently engaging in less than 150 min of MVPA per week [38]. There were no inclusion or exclusion criteria specific to RT. Participants were excluded from the study if they reported any contraindications to exercise or had recent or planned surgery (including reconstructive surgery). However, there were unplanned surgeries throughout the trial, including during this RT study extension. The trial was conducted at the University of Toronto (Ontario, Canada). Ethical clearance was received from the University of Toronto’s Human Research Ethics Unit (#00038665). The reporting of this study conforms to the STROBE statement [39].

### 2.2. Design and Recruitment

Recruitment for the C4E trial was originally completed through digital materials, including e-mails to community centers and social media posts which signaled interested participants to contact the study team. Participants in the original study included 108 WBC, of which 45 were recruited to the RT extension based on self-selection. The post hoc power calculation in G*Power [40] for α = 0.05, correlated means and small-to-moderate effect size with 44 participants suggests 83% power has been achieved for a one-tailed test (i.e., improvements in outcomes). All participants received a Fitbit, an ’Exercise Peer Support Guide’, and a one-page infographic highlighting current exercise guidelines for cancer survivors in the original study. There were three assessments during the current 8-week RT intervention, which ran from May to July 2022: (i) demographics and baseline (pre-intervention; T1), (ii) post-intervention (8 weeks post-baseline; T2), and (iii) final post-intervention follow-up (approximately 20 weeks post-baseline; T3). Sources of bias include the self-report surveys used for assessments (i.e., social desirability, recall) that were reduced by using validated and reliable scales, as well as selection bias, as participants were self-selected which may bias the intervention effect. Objective measures for muscular strength were conducted virtually with a trained health professional using appropriate guidelines to reduce bias. 

### 2.3. Intervention 

Participants were matched with a peer to provide social support. Re-matching women with new women helped reduce any biases that may have been introduced with quality of original matches, connections, and familiarity. Matches were made both in the original C4E and in this RT study using evidence-based criteria [17,41], including age, family status (children), current physical activity level, and time zone. An information session was held to introduce participants with their peers, discuss the study, review safety measures, and answer questions. The session was recorded and sent to all participants. Participants were given up to $40 CAD each to purchase equipment, although it was made clear that equipment was not mandatory. 

Participants received the option of four QEP-led Zoom RT session times per week to accommodate different schedules. The participants chose their preferred time (regardless of their partners’ preferred time) for one weekly QEP-led Zoom RT session. Participants were also instructed to meet online with their partner on a separate day during the same week to complete the same weekly workout they performed with the QEP. 

The intervention design was informed by the Capability, Opportunity, Motivation, Behaviour (COM-B) model [2,33]. The COM-B model is often used in the development of interventions aimed at increasing physical activity [34]. This theoretical framework suggests that capability (perceptions of ability to engage in behavior), opportunity (environmental aspects that enable behavior), and motivation (reflective and automatic processes that facilitate or restrict behavior) are required to influence behavior change [32]. The aspects of the intervention that align with each component of the COM-B model are outlined in Appendix A: Table A1. In general, QEP-led sessions and weekly educational components were used to target capability, virtual session offerings, and peer matching, group sessions were used to address opportunity, goal setting, workout logs, and behavior modeling (group and peer sessions), and weekly educational components were used to target motivation. Weekly QEP-led Zoom sessions lasted 60–75 min and consisted of (a) a 10-min informational discussion and (b) 50–60 min of RT. Participants were emailed the weekly session workouts every Sunday, which included video and written directions. Each workout consisted of breathwork, a warmup, main exercises (i.e., a squat/lunge pattern, a push, a pull, a hip extension, a hinge, core work), and mobility work, which was considered the cooldown (see Appendix A: Table A2 for an example). The weekly workout was modeled after the previous week, to emphasize weekly progression, where participants were advised to try to progress their movement patterns, if possible. Progression was defined as working on exercise form, increasing the weight, increasing the reps/sets, and changing exercise tempo [42,43]. Each week, there was a specific focus on different types of progressions (for example, in week 2, increased reps were encouraged compared to the previous week). In addition to the weekly RT workouts, the 10-min discussion involved information before the workout began, with topics such as rest and recovery, progressive overload, designing your own program, and body functionality appreciation (i.e., the appreciation, respect, and honor of your body for its functions) [44], among others (see the Appendix A: Table A1 for a summary of the topics). The muscular strength tests were administered during the first and last week of the QEP-led Zoom RT sessions. For data collection and accountability purposes, each participant also filled out an online workout accountability check-in by self-reporting their weights/reps/sets for each QEP-led session and partner session, if applicable. 

### 2.4. Data and Safety Monitoring

The lead QEP received advanced training on exercise for individuals living with and beyond cancer, developed specifically for health and fitness professionals (Thrive Health Services Ltd.). Appropriate training and certification in research ethics helped to limit the introduction of biases in leading the sessions. Two additional QEPs, also appropriately trained in exercise delivery and research ethics, were involved in supporting roles. Before the trial, participants were screened for health and safe exercise using the participant intake questionnaire. A safety team consisting of the principal investigators, QEPs, and the study coordinator met weekly to discuss participant medical issues and compliance. These compliance data meetings reduced study bias by having standardized procedures for the intervention and increased transparency between the lead QEP and the study team, which ensured consistency and minimized variability that could introduce bias into the results. In addition, meetings were planned as needed and any adverse events were reviewed and addressed. During the QEP-led Zoom RT sessions, a minimum of two QEPs and one research assistant were online to monitor the safety of all participants and were able to give feedback to individuals during the workout. Before the sessions, the lead QEP verbally emphasized safety practices during physical exertion, such as staying hydrated, recognizing signs of fatigue, and pacing oneself during the workouts. The original information session also reviewed safety measures for exercise. Participants also had a weekly online check-in form to report any adverse events and these events were reviewed by the QEP prior to the sessions. 

### 2.5. Measures

#### 2.5.1. Descriptive Data and Covariates

Participant demographics (e.g., age in years, gender, ethnicity, relationship status, number of children, education, primary language, employment status, and time zone) and cancer-related demographics (e.g., stage of cancer, time since diagnosis, treatment status, treatments undergone and number of medications) were reported during T1. Attendance was taken at each QEP-led Zoom RT session, and participants reported if they completed their suggested weekly partner workout during their online accountability check-in. 

#### 2.5.2. Feasibility Assessments

**Enrolment:** enrolment rate was assessed as the percentage of eligible WBC who participated in the original C4E intervention that enrolled in the present intervention.

**Adherence:** Adherence to QEP-led sessions was measured by recorded attendance. Adherence to partner sessions was measured by self-reported completion. In addition, participants were asked two open-ended questions regarding communication, feedback, and challenges attending the QEP and partner sessions in T2; (i) “Please comment on what made attending the (QEP-led zoom RT) session difficult or easy” and (ii) “What, if anything, made it difficult to communicate with or relate to your exercise partner?”.

**Retention:** retention rate was measured as the percentage of enrolled participants who completed post-intervention and follow-up assessments. 

**Safety:** Participants were asked to report any adverse events that occurred during the intervention by contacting the research team. Safety was assessed by the number and type(s) of adverse events participants reported that were related to the intervention.

**Social support:** Social support was assessed using both peer match perceptions and communication. Participants’ match satisfaction, quality, and similarity were explored by asking participants to respond to several questions at T2. Match satisfaction was measured using one item: “How satisfied are you with the quality of your exercise partner match to support exercise in this project?” from 1 = very dissatisfied to 4 = very satisfied. Match quality was assessed using one item: “To what extent is/was your assigned exercise partner a good match for you?” from 1 = very poor match to 4 = very good match. Finally, match similarity was measured using one item: “To what extent is/was your assigned exercise partner a good match for you?” from 1 = not at all similar to 4 = extremely similar. As an additional social support metric, participants were asked to report at T2 and T3 how often they communicated with their partner each week (on average). Participants reported an average number of times per week (open-ended). In addition, participants reported how they communicated (i.e., e-mail, text, instant messaging, phone call, video call, in person and other). Participants could check all that applied.

**Therapeutic Alliance:** Therapeutic alliance was measured at T2 using a 16-item modified Working Alliance Inventory (WAI) [21], which assesses the collaborative relationship between the lead QEP and the participants. This modified version specified that the therapist (in the original wording of the scale) is the physical activity trainer and asked them to describe how often they feel the response choice scenarios happened during their QEP-led Zoom RT sessions (e.g., “My physical activity trainer and I agree on what is more important for me to work on” from 1 = never to 7 = always). Appropriate items were reverse-coded, and the scores were summed and averaged, wherein a higher average score represents greater therapeutic alliance. Evidence of reliability is previously demonstrated in WBC in the context of their relationship with their surgeons [45] and in adults in the context of evaluating the personal trainer–client relationship [46].

#### 2.5.3. Outcome Assessments

**RT:** RT volume, measured by total weekly minutes of RT, was calculated using the modified Godin Leisure Time Exercise Questionnaire (GLTEQ) [47] at each time point (T1–T3). Participants were asked to report their weekly minutes of RT activities, which was summed for a weekly RT score (average minutes per week). The weekly reported RT days per week were used to calculate the meeting guidelines of ≥ 2 days of RT per week (yes, no) [3,6]. 

**MVPA:** Exercise volume measured by total weekly MVPA was calculated using the modified GLTEQ [47] at each time point (T1–T3). Participants were asked to report their weekly minutes of moderate and strenuous activities, which were summed for a weekly MVPA score (average minutes per week). The weekly MVPA score variable was used to calculate the meeting guidelines of ≥150 min of MVPA per week (yes, no) [3,6]. MVPA was examined because of its importance to health and well-being [3,6]. The modified GTLEQ has been used with WBC [48], has demonstrated validity [49], and test–retest reliability has been established in general adult samples [47].

**Guidelines:** Meeting-combined MVPA and RT guidelines were calculated (yes, no) [3].

**Muscular strength:** For lower body strength, the Five times sit-to-stand (5TSTS) was performed at the first (week 1) and last (week 8) QEP-led Zoom RT session. The score is the amount of time (to the nearest decimal in seconds) it takes a participant to transfer from a seated to a standing position and back to sitting five times with their arms crossed in front of them. After a brief explanation and demonstration of the evaluation, checking for safety and ensuring all participants were ready online, one QEP started the screen clock while the other started the procedure. Participants recorded their times and sent them to the study coordinator during their online weekly accountability check-in. Evidence of reliability is previously demonstrated in WBC [50,51]. 

For upper body strength, the bicep arm curl test was performed at the first (week) and last (week 8) QEP-led Zoom RT session. The goal is to perform as many repetitions of the arm curl as possible in 30 s using the dominant arm. The arm curl test is usually carried out with a 5-pound weight for women and an 8-pound weight for men [52]; however, we instructed the participants to use the weight they had available, and used total volume as our measurement (weight × reps), as not all participants had 5-pound dumbbells. Participants start with their arm hanging down beside a chair and curl the dumbbell up towards their biceps and then return to a fully extended position and count the total number of reps performed in 30 s. After a brief explanation and demonstration of the evaluation, checking for safety and ensuring all participants were ready online, one QEP started the screen clock while the other started the procedure. Participants recorded their number of bicep curls and the weight they used and sent this information to the study team during their online weekly accountability check-in. Evidence of reliability of the bicep arm curl test is previously demonstrated [53] and it has been used in exercise trials with WBC [54].

**The RT Self-Efficacy Scale:** The modified 10-item exercise self-efficacy scale (ESES) [55] is a scale developed to measure beliefs or confidence in performing various physical activities and exercise. This modified version specifies that the exercise is RT. Participants answered how confident they were in carrying out regular RT (e.g., “I am confident…that I can overcome barriers and challenges with regard to RT if I try hard enough”) on a 4-point scale (1 = not always true to 4 = always true). The scores were summed and averaged, wherein higher scores reflect greater self-efficacy for RT. Evidence of reliability is previously demonstrated in adult women with obesity [56].

**Physical Self-Perceptions:** The 24-item Physical Self-Description Questionnaire (PSDQ) short form [57] includes seven subscales assessing perceptions of physical activity, appearance, body fat, coordination, flexibility, and strength, as well as global physical self-concept. Participants rated items on a 6-point scale from 1 (false) to 6 (true), with higher scores reflecting more favorable physical self-perceptions. The reliability of this instrument has been demonstrated with non-cancer older adult populations [57].

### 2.6. Data Analysis

***Preliminary Analysis:*** The data were screened for normality, outliers, and missing data (see Appendix A: Table A3 for the missing data description per variable). Descriptive data for continuous variables were reported as means and standard deviations (SDs), and categorical variables were reported as frequencies and percentages. The distribution of the study variables (e.g., testing variable distribution) was assessed through a histogram, and skewness and kurtosis values and McDonald’s omega (ω) reliability coefficients [58] were computed for study variables. Where appropriate, complete case analyses were conducted and missing data were not replaced. There were no post hoc subgroup analyses nor any sensitivity analyses computed.

***Main Analysis:*** To address the main feasibility aims 1a (enrolment rate), 1b (adherence rate), 1c (retention rate), 1d (adverse events reported), 1e (participants’ ratings of their peer-match post-intervention), 1f (participant’s communication frequency and mode of communication with their peer match post-intervention and follow-up), and 1g (participants’ ratings of the therapeutic alliance with the QEP post-intervention), descriptive data for continuous variables were reported as means and standard deviations (SDs), and categorical variables were reported as frequencies and percentages.

To address secondary aims 2a and 2b (change in RT and MVPA from baseline to post-intervention and follow-up), separate one-way repeated measures ANOVA with post hoc pairwise comparison (when applicable) was used to compare mean differences in weekly minute of RT and MVPA from T1, T2, and T3. To address secondary aim 2c [change in strength (assessed through the sit-to-stand test and bicep curl test) from baseline to post-intervention], paired-samples *t*-tests were conducted on the 5TSTS and bicep curl test from T1 to T2. To address secondary aim 2d (change in RT self-efficacy from baseline to post-intervention and follow-up), a one-way repeated measures ANOVA with post hoc pairwise comparison (when applicable) was used to compare mean differences in ESES from T1, T2, and T3. To address the tertiary aim 3a (change in physical self-perceptions from baseline to post-intervention and follow-up), separate one-way repeated measures ANOVA with post hoc pairwise comparison (when applicable) was used to compare mean differences in physical self-perceptions from T1, T2, and T3. Finally, to address 3b (the relationship between changes in RT behavior and possible changes in physical self-perceptions, ESES, MVPA, 5TSTS and bicep curl test), Pearson’s bivariate correlations were computed between change score variables from T1 and T2. Bonferroni adjustments were applied to correct for multiple comparisons by multiplying the uncorrected *p*-value by the number of comparisons, with statistical significance set at Bonferroni-corrected *p*-values of ≤ 0.05. Cohen’s *d* was used to observe effect sizes as small, medium, or large using 0.20, 0.50, and 0.80, respectively. Analyses were conducted in IBM Statistical Package for the Social Sciences (SPSS) version 26. Statistical significance was set at *p* < 0.05.

## 3. Results

Of the 108 women from the original C4E intervention, 45 provided informed consent for the RT intervention and data for T1. As the intervention progressed, 41 participants completed T2, and 39 participants completed T3. Due to study drop out, the analytic sample comprised of 41 participants. 

### 3.1. Descriptive Data 

The age of the 41 participants retained for analysis was *M*_age_ ± SD = 50.7 ± 11.2 years. See Table 1 for sociodemographic and clinical characteristics. 

### 3.2. Feasibility 

***Enrolment:*** Of the 108 WBC who participated in the original intervention, 41.6% enrolled in the present intervention.

***Adherence:*** For the 8-week intervention, attendance rate for the QEP sessions was 63.8%, with 40.0% for the peer session. Participants attended the weekly QEP-led Zoom RT sessions with a mean (SD) of 5.1(2.3) (range = 0–8) and a mean (SD) of 3.2(2.3) (range = 0–7) sessions per week with their study partners. See Appendix A: Table A4 for participant feedback on the facilitators and challenges they reported when attending the QEP-led and partner sessions. Overall, reasons for not attending the weekly QEP-led sessions included physical (e.g., illness, surgery), environmental (e.g., travel, work, program level of difficulty), and psychosocial (e.g., differing goals, perceived time limits). Meanwhile, reasons for not attending partner sessions included physical (e.g., time zone differences), environmental (e.g., work schedule), and psychosocial (e.g., differing goals, not having a facilitated introduction). Many participants enjoyed the virtual format and stated reasons such as the flexibility of the QEP sessions offered, allowing them to spend more time with their family (rather than going to a fitness center). They felt that their environment was safe and that the QEPs were effective and qualified, they enjoyed the extra virtual material sent (e.g., YouTube videos) and having a set time was a motivator to schedule the workouts and adhere to them. Many participants stated that having many virtual time options was ideal.

***Retention:*** Of the 45 WBC who enrolled in the intervention, 91.1% completed post-intervention assessments (T2) and 86.6% completed follow-up assessments (T3).

***Safety*:** No adverse events were reported.

***Social Support: Participant Match Satisfaction, Quality, and Similarity***: Participants reported a mean (SD) of 3.2(0.9) (range = 1.0–4.0), 3.0(1.0) (range = 1.0–4.0) and 2.8 (1.0) (range = 1.0–4.0) for match quality, match satisfaction, and match similarity, respectively. 

***Social Support: Participant Match Communication***: Participants communicated a mean (SD) of 1.3(1.1) times per week (range = 0–5) via text message (50%), e-mail (33%), video call (29%), phone call (17%), Facebook messenger (12%), and in person (2%) during the intervention (weeks 1–8). Post-intervention, 43% of participants reported still being in communication with their study partner at T3 and communicated a mean (SD) of 1.2 (0.8) times per week (range = 0–2). Of the 43% (*n* = 17 of 39) who were still in communication with their partner post-intervention, 47% (*n* = 8 of 17) reported continuing with the partner exercise sessions.

***Therapeutic Alliance***: The WAI demonstrated internal consistency in T2 (ω) = 0.96. Participants reported a mean (SD) of 5.8(1.2) (range = 1.75–7.0) on the modified WAI. 

### 3.3. Secondary and Tertiary Aims

***RT***: There were significant differences in total weekly minutes of RT over the three time points (*F*(2, 70) = 13.77, *p* < 0.001). See Table 2 for the means and SDs per time point. Post hoc analysis with a Bonferroni adjustment revealed that total weekly minutes of RT significantly increased pre- to post-intervention (41.5 (95% CI, 20.6 to 62.4) min/week, *p* < 0.001, *d* = 0.82 between T1 and T2 scores), and significantly decreased post-intervention to the final follow-up (−25.3 (95% CI, −43.8 to −6.73) min/week, *p* = 0.005, *d* = 0.48 between T2 and T3 scores) yet remained significantly higher than baseline (16.2 (95% CI, 10.15 to 50.18) min/week, *p* = 0.002, *d* = 0.28 between T1 and T3 scores). Participants reported meeting RT guidelines 31.3%, 72.5% and 43.2% in T1, T2 and T3, respectively.

***MVPA***: The total weekly minutes of MVPA did not differ significantly across the three time points (*F*(2, 58) = 1.35, *p* = 0.27) (see Table 2). The effect sizes for change in MVPA from T1 to T2, T2 to T3, and T1 to T3 were *d* = 0.21, 0.22, and 0.02, respectively. Participants reported meeting MVPA guidelines 27.5%, 32.4% and 26.3% in T1, T2, and T3, respectively. 

***Guidelines:*** Participants reported meeting both MVPA and RT guidelines in T1, T2 and T3 20.0%, 22.0% and 21.6%, respectively.

***Muscular strength:*** Lower body strength (5TSTS) improved significantly, with a decrease in time to stand from T1 to T2 (*t*(1, 30) = −2.94, *p* = 0.006, *d* = 0.041) (Table 2). Upper body strength (total bicep curl volume) increased significantly from T1 to T2, (*t*(1, 30) = 4.45, *p* < 0.000, *d* = 0.82) (Table 2).

***The RT Self-Efficacy Scale (ESES-modified)***: No significant differences over the three time points (*F*(2, 30) = 0.71, *p* = 0.49, *d* < 0.05) were observed for the ESES (Table 2). 

***The Physical Self-Description Questionnaire (PSDQ):*** There were significant differences in appearance self-perceptions (*F*(2, 72) = 3.84, *p* = 0.02), and general physical self-concept (*F*(2, 72) = 4.39, *p* = 0.02), over the three time points; see Table 3. Appearance self-perceptions significantly increased from post-intervention to follow-up (0.48 (95% CI, 0.01 to 0.95), *p* = 0.04, *d* = 0.61 between T2 and T3 scores), and general physical self-concept significantly increased from baseline to post-intervention (0.69 (95% CI, 0.06 to 1.34), *p* = 0.03, *d* = 0.46 between T1 and T2 scores).

Change in minutes of RT from baseline to post-intervention was significantly positively correlated with change in activity self-perception (*r* = 0.62, *p* < 0.001; Table 4), with all self-perceptions correlated at small-to-moderate effect sizes with change in RT. Changes in RT were not significantly correlated with changes in the ESES (*r* = 0.22, *p* = 0.18), changes in MVPA (*r* = −0.02, *p* = 0.92), changes in bicep curl test (*r* = 0.16, *p* = 0.39) and the 5TSTS (*r* = −0.30, *p* = 0.10).

## 4. Discussion

This pilot single-arm pre–post intervention study aimed to examine the feasibility of an 8-week virtually delivered and socially supportive RT intervention for WBC and to test changes in physical activity-related variables. Overall, the pilot intervention was feasible (e.g., high retention rate, satisfaction with peer and QEP social support), safe (i.e., no adverse events reported), and demonstrated effectiveness for increasing weekly time spent RT, muscular strength, and activity self-perception. The results are encouraging, as they provide preliminary evidence for using virtual socially supportive RT interventions for WBC.

The present study had an enrolment rate of 42%, which aligns with previous peer-based physical activity interventions for WBC [23] and indicates a high level of RT interest among WBC. Researchers designing future RT interventions for WBC should consider increasing the recruitment period and varying the ways in which the study information is communicated to potential participants [59]. For example, an emphasis on the safety of RT is recommended in recruitment messaging, as this is often a concern among WBC [60]. In terms of retention rates, 91% of women were retained post-intervention and 87% remained at follow-up, which is high yet slightly lower when compared to previous virtually delivered RT interventions [12] and peer-based physical activity interventions [23] for WBC. Compared to other trials that are often focused on WBC post-treatment, the current sample included women in active treatment and across the full spectrum of stage of breast cancer, and these characteristics may explain nuanced differences in retention rates. To optimize retention rates in future RT interventions for WBC, researchers may consider more interaction in virtually delivered sessions (e.g., process-related goals and challenges, regular goal setting, self-reflection, action planning and implementation intentions). Finally, researchers could consider further incentivizing participants post-enrollment [61]. 

Adherence, as assessed by attendance in QEP sessions and partner sessions, was 64% and 40%, respectively. While the QEP adherence rate generally falls within the adherence range of other remotely delivered physical activity interventions for cancer survivors [11], improvements are needed. Commonly reported reasons for missing QEP were travel and scheduling issues (e.g., childcare, summer travel) and increased flexibility in session offerings may improve adherence. Women may benefit from self-regulation to adjust RT goals when personal and family responsibilities change [62]. The adherence rate was especially low for partner-based sessions, with WBC commonly reporting barriers of different time zones and schedules. It may be that the autonomous nature of the partner sessions (i.e., the women scheduled the sessions themselves) was premature, and the intervention team may be an important source for facilitating adherence to partner sessions. For example, facilitating and “booking” the partner sessions and addressing missed sessions as a group may have increased the adherence rates. Overall, engaging women in discussions about adherence and exploring strategies to improve attendance is important for optimizing intervention outcomes.

In general, assessments of the social support aspects of the program revealed that WBC were satisfied with the peer match and QEP support. WBC reported feeling their assigned partner was a good match, similar to them, and were satisfied with the quality of their exercise partner match. Most interventions do not solicit perceptions of peer match quality [17] and this information provides insight into the appropriateness of our matching criteria. Partners reported communicating approximately once per week during the intervention, most commonly via text message, email, and video calls, and 43% of women continued communication with their partner. This cadence of communication is comparable to other peer-based physical activity interventions [17,23], yet investigating the optimal frequency for peer communication would be a worthwhile avenue of future research. Further, aligned with previous recommendations [17], future work examining the optimal mode of peer communication is needed. 

Related to the QEP, WBC reported high therapeutic alliance, indicating they were satisfied with the connection generated with the QEP and felt they agreed with the QEP regarding their goals [21]. Given the importance of quality social support and perceived safety of WBC for intervention adherence and increased physical activity behavior [15,17], it is recommended that future virtually delivered RT interventions for WBC continue to offer high quality QEP support. Importantly, QEPs working with WBC should have advanced training on exercise for people with cancer and knowledge of optimal behavior change strategies. This tailored training may have contributed to the quality of trainer–participant relationships and the absence of adverse events reported in the present study. Further feedback on QEP support was not gathered, and researchers are encouraged to incorporate qualitative methods in future RT interventions to gather participants’ in-depth perceptions on their experiences working with QEPs. 

Aligned with our hypothesis, WBC increased their RT from baseline to post-intervention with a large effect size. In fact, 72.5% of participants were meeting RT guidelines [3,6] post-intervention compared to 31.3% pre-intervention. However, RT was not fully sustained at follow-up, with 43.2% of WBC meeting RT guidelines twelve weeks post-intervention. These results mirror many physical activity interventions that do not effectively sustain physical activity improvements over time [63]. Researchers are encouraged to consider the support participants may need to maintain RT post-intervention. Teaching participants how to use behavior change techniques (e.g., how to plan future RT sessions) may support RT maintenance [63]. For example, McEwan and colleagues [63] suggest that targeting the behavior change technique of self-beliefs (i.e., building confidence in abilities to perform physical activity) may help sustain physical activity improvements. In the present study, participants’ RT self-efficacy did not significantly increase over time, suggesting that self-beliefs were not adequately fostered. Implementing strategies to increase RT self-efficacy (e.g., self-talk, emphasize past successes) should be considered when examining this RT intervention in future work. 

Contrary to the previous literature [13,15,17] and our hypothesis, the 23 min per week increase in MVPA during the intervention was not significant and equates to a small effect. Nonetheless, this intervention was not designed to increase MVPA, and participants may have been focused on engaging in RT at the expense of MVPA. When implementing RT-only interventions, researchers should consider providing education around the importance of also increasing MVPA with participants. As an additional metric of physical fitness, WBC improved their strength from baseline to post-intervention, with medium to large effect sizes for upper and lower body indicators. This result is aligned with interventions that used combined aerobic and RT protocols [11,13] and RT-only interventions delivered to individuals with mixed cancer diagnoses [14]. These enhancements in muscle strength have important clinical implications. Physically, enhanced muscular strength contributes to long-term health outcomes, such as reduced risk of fractures [64], and potential prevention of cancer recurrence [65]. Functionally, the increase in strength may afford WBC better control over everyday activities, potentially reducing the risk of injury and enhancing quality of life [5]. Psychologically, the empowerment derived from increased physical capabilities can foster a positive self-concept and contribute to mental well-being [66]. 

Reflecting prior research on RT and physical self-perceptions in women [31], the pilot data indicated that women improved their appearance self-perceptions and general physical self-concept, and there was a relationship between increased RT engagement and significantly improved activity self-perceptions. Additionally, RT improved other important self-perceptions with small-to-medium effects, including appearance and body fat perceptions, that are integral to overall body image [67]. The enhanced self-perceptions and activity self-concept could potentially promote an active lifestyle by creating a reinforcing cycle where physical activity strengthens the physical and active self-concept, encouraging further physical engagement [28]. The additional changes in self-perceptions require further attention and may be important in sustaining RT long term.

There are limitations to this study that warrant consideration. Our study results are limited to those who met the study inclusion and exclusion criteria, reducing external validity. For example, only females diagnosed with breast cancer were eligible to participate in the study and more work is needed to include individuals of other sex and gender identities. The lack of a control group precludes our ability to definitively conclude that the significant changes were due to the intervention. Researchers are encouraged to test this intervention with a larger sample size and a control group. Adherence was examined based on the number of sessions of RT but should also be examined as compliance with program elements in further work. Finally, there was no specific nutrition component within the intervention, and WBC could also benefit from lifestyle behaviors including but not limited to diet, sleep, substance use, and sun safety.

Despite the limitations, this study had several strengths worth noting. The intervention components were informed by the COM-B model [32] and the RT sessions were designed to offer individualized progressions. No adverse events were reported, and there was a high retention rate. The virtual format of the intervention also removed several reported barriers to physical activity [7,8,9] and provided access for those who were unable to attend programs in person. Finally, social support was fostered through group-based QEP-led RT sessions and matching WBC to peers for support and an additional paired RT session. These socially supportive features strengthen the current pilot intervention, as they address physical activity barriers reported by WBC (i.e., access to QEP support, finding a peer exercise partner) [7,8]. 

## 5. Conclusions

In summary, this pilot single-arm pre–post RT intervention study for WBC was feasible, safe, and demonstrated preliminary evidence for increasing RT time, strength, and activity self-perception. To our knowledge, this is the first virtually delivered RT-only intervention for WBC that was designed to leverage social support. This intervention may improve quality of life among WBC by managing the acute and lasting effects of breast cancer, and therefore should be rigorously examined in future research. Further research is needed to explore long-term effects, assess scalability, and compare virtual interventions with more traditional in-person RT programs to establish a comprehensive understanding of benefits and optimization for implementation in oncology care.

## Figures and Tables

**Table 1:** Selected sociodemographic and clinical characteristics of participants in the resistance training intervention.

Variables	*n*	*M* (SD) or %
**Sociodemographic characteristics**		
Age (M(SD))	41	50.7 (11.2)
Gender (% women) ^a^	40	97.6%
Ethnicity (% Caucasian/White)	29	70.7%
Married or living with partner (% yes)	29	70.7%
Children (% yes)	29	70.7%
Highest level of education completed (% ≥ university)	36	87.7%
Household income (% ≥ 100,000 CAD)	20	48.8%
Primary language (% English)	37	90.2%
Currently employed, full- or part-time (% yes)	16	39.0%
Time zone (% yes)		
Central	1	2.4%
Eastern	34	82.9%
Mountain	2	4.9%
Pacific	4	9.8%
**Health and cancer-related characteristics**		
Breast cancer stage diagnosis (% Stage ≥ II)	25	60.9%
Treatment status (% currently receiving treatment)	5	12.2%
Number of medications (M(SD))	41	1.7 (1.6)
Years since diagnosis (% after 2018)	26	63.4%
**Breast cancer treatment (% yes)**		
Lumpectomy	21	51.2%
Single or double mastectomy	22	53.7%
Chemotherapy	31	75.6%
Radiotherapy	29	70.7%
Hormonal therapy	18	43.9%

Note. ^a^ One participant reported being non-binary.

**Table 2:** Means, standard deviations and percentages for physical activity measures used in the resistance training intervention.

Variables	T1 *M* (SD), or %	T2 *M* (SD), or %	T3 *M* (SD), or %
Total minutes of RT/week	26.3 (34.3) ^a,b^	67.8 (48.6) ^c^	42.5 (50.3)
Meet RT guidelines, %yes	31.3	72.5	43.2
Total minutes of MVPA/week	87.4 (81.8)	110.3 (101.9)	84.9 (70.7)
Meet MVPA guidelines, % yes	27.5	32.4	26.3
Meet MVPA and RT guidelines, % yes	20.0	22.0	21.6
5TSTS	8.3 (2.3) ^a^	7.3 (2.4)	-
Bicep curl volume	58.6 (34.0) ^a^	83.9 (32.7)	-
ESES	2.9 (0.6)	3.0 (0.6)	2.9 (0.8)

Note: **^a^** T1 differs significantly from T2; **^b^** T1 differs significantly from T3; **^c^** T2 differs significantly from T3. MVPA: moderate to vigorous physical activity; RT: resistance training; 5TSTS: Five times sit-to-stand; ESES: resistance training self-efficacy scale

**Table 3:** Means and standard deviations for physical self-perception measures during the resistance training intervention.

Variables	T1 *M* (SD)	T2 *M* (SD)	T3 *M* (SD)
Activity SP	2.8 (0.19)	3.3 (0.15)	3.0 (0.19)
Appearance SP	3.9 (0.15) ^b^	3.5 (0.16)	3.9 (0.12)
Body Fat SP ^c^	2.7 (0.24)	3.1 (0.27)	3.1 (0.22)
Coordination SP	4.4 (0.15)	4.5 (0.17)	4.5 (1.5)
Flexible SP	3.7 (0.18)	4.0 (0.18)	4.1 (0.16)
Physical self-concept	3.2 (0.17) ^a^	3.9 (0.17)	3.7 (0.18)
Strength SP	3.6 (0.91)	4.0 (1.1)	4.0 (1.0)

Note. SP = self-perceptions; **^a^** T1 differs significantly from T2; **^b^** T2 differs significantly from T3; **^c^** Body fat SP is reverse-scored such that higher scores reflect perceptions of less body fat.

**Table 4:** Descriptive statistics, Pearson’s bivariate correlations (95% confidence intervals), and internal consistency for change in resistance training pre–post intervention and physical self-perceptions.

Variables	1	2	3	4	5	6	7	8
1. Change in RT ^a^	-							
2. Activity SP	0.62 ** (0.39, 0.78)	-						
3. Appearance SP	0.29 (−0.02, 0.55)	0.45 ** (0.16, 0.66)	-					
4. Body fat SP ^b^	0.25 (−0.06, 0.52)	0.28 (−0.04, 0.54)	0.33 * (0.02, 0.58)	-				
5. Coordination SP	0.16 (−0.16, 0.44)	0.27 (−0.04, 0.53)	0.65 ** (0.43, 0.80)	−0.08 (−0.38, 0.24)	-			
6. Flexible SP	0.15 (−0.17, 0.43)	0.27 (−0.04, 0.54)	0.47** (0.19, 0.68)	0.25 (−0.06, 0.52)	0.59 ** (0.34, 0.76)	-		
7. Physical self-concept	0.12 [−0.20, 0.41]	0.31 (−0.01, 0.56)	0.74 ** (0.56, 0.85)	0.33 * (0.03, 0.58)	0.62 ** (0.39, 0.78)	0.51 ** (0.24, 0.71)	-	
8. Strength SP	0.16 (−0.16, 0.44)	0.29 (−0.02, 0.55)	0.67 ** (0.46, 0.81)	0.15 (−0.17, 0.44)	0.86 ** (0.75, 0.92)	0.56 ** (0.31, 0.74)	0.65 ** (0.43, 0.80)	-
Mean (SD) at T1	29.5 (36.8)	2.8 (1.2)	3.5 (0.9)	2.6 (1.5)	4.4 (0.9)	3.7 (1.1)	3.2 (1.0)	3.7 (0.9)
Mean (SD) at T2	70.1 (50.8)	3.3 (1.0)	4.0 (1.0)	3.0 (1.6)	4.5 (1.0)	4.0 (1.1)	4.0 (1.1)	4.1 (1.1)
Cohen’s *d* [95% CI] ^c^	**0.74** **(−1.1, −0.39)**	0.33 (−0.64, −0.01)	0.31 (−0.62, 0.01)	0.20 (−0.51, 0.11)	0.08 (−0.38, 0.23)	0.21 (−0.52, 0.10)	**0.47** **(−0.79, −0.15)**	0.24 (−0.55, 0.07)
Scale range	-	1–6	1–6	1–6	1–6	1–6	1–6	1–6
Internal consistency (ω) for T1 and T2, respectively	-	0.87, 0.89	0.86, 0.84	0.71, 0.68 ^d^	0.93, 0.95	0.89, 0.89	0.83, 0.90	0.84, 0.90

Note. All correlations computed with change scores from baseline to post-intervention; SD = standard deviation; T1 = baseline; T2 = post-intervention; CI = confidence intervals; ω = McDonald’s omega. SP = self-perceptions. ^a^ Change in RT = change in total minutes spent resistance training per week from baseline to post-intervention. ^b^ Reverse coded; subscale comprised of waist and overweight items only. ^c^ Paired-samples *t*-tests for change in T1 and T2. Based on Bonferroni corrections, bolded values demonstrate significant values (p ≤ 0.05). Cohen’s *d* guidelines: small = 0.2; medium = 0.5; large = 0.8. ^d^ Pearson’s (*r*) bivariate correlations between the two body fat self-perception items at baseline (95% CI = 0.51, 0.83) and post-intervention (95% CI = 0.47, 0.82), respectively. * Significant at the *p* < 0.05 level ** Significant at the *p* < 0.01 level.

## Data Availability

The datasets generated and analyzed during the current study are available from the corresponding author on reasonable request.

## Supplementary Materials

The following supporting information can be downloaded at: https://www.mdpi.com/article/10.3390/cancers16162829/s1, File S1: CONSORT 2010 checklist of information to include when reporting a pilot or feasibility trial.

## Appendix A

**Table A1:** Intervention design factors using the COM-B model.

Capability	Opportunity	Motivation
Zoom RT QEP-led sessions were offered four times a week during am, pm, and evening to accommodate schedules	Flexible group class option for the QEP session (offered up to four times a week)	Social support through QEPs, study partner and group Zoom RT sessions
QEP broke down and demonstrated all exercises and offered 3–5 alternatives	Virtually delivered QEP-led Zoom sessions	Weekly encouragement, feedback and support were given through e-mail as well as feedback by two QEPs given after each RT session
Two QEPs and one research assistant were online during all sessions to support and observe	All session plans were sent weekly on Sundays via e-mail with video and written directions before the weekly sessions for review	Received a social support guide to support partner relationship
Proper warm-ups and cool-downs were offered each session	Social support opportunity through QEP and Peer	Set weekly goals as a group
Breathwork offered each session	Offered many exercise alternatives, and gave autonomy to choose the alternative or omit the exercise	Used online workout log to track all repetitions/sets
QEPs remained online after sessions to discuss questions and go over exercise form with each participant (as needed)	Choice of partner workout day	Weekly educational component during QEP RT sessions *
Gradually used progressive overload each week **	Testing strength before and after	Gradually used progressive overload each week **
Weekly educational component during QEP RT sessions *	Pre-study information session	Tested strength before and after intervention
Self-monitoring offered through the Fitbit and weekly exercise accountability self-report online forms	Addition “accessory” work suggestions were given for those who were interested (in the weekly e-mail)	Given a study fitness tracker (Fitbit) for accountability and awareness
	Participants were given $40 CAD toward equipment	Participant administered Facebook support group provided post-intervention for all participants

* One topic was covered per week. Topics included RT program principles, key terms in RT, mind–muscle connection, setting yourself up for success, body functionality appreciation, rest and recovery, mobility work, and how to continue weight training after the sessions are over. PowerPoints for content available upon request. ** Through emphasis on form, increasing weight/repetitions/sets, using tempo. QEP: qualified exercise professional; RT: resistance training.

**Table A2:** Example of the weekly workout (all webs accessed on 7 August 2024; the * is a disclaimer).

C4E Workout Week 1				
Exercise Name	Options	Sets	Repetitions	Link to Demonstration/Options
Squat	chair squat, supported chair squat, band, with weights, barbell, seated leg extension, body weight, lateral band walk, donkey kick, stability wall squats, banded, calf raises, seated leg lifts	2	8–12	https://exrx.net/WeightExercises/Quadriceps/BWSquat
Shoulder press	standing, seated, body weight, lateral raises, single arm, banded	2	8–12	https://exrx.net/WeightExercises/DeltoidAnterior/DBShoulderPress
Single arm row	seated row, banded	2	10–12 (each arm)	https://exrx.net/WeightExercises/BackGeneral/DBBentOverRow
Bicep curl	seated curl, wall push-up, push-up (any modification), single arm	2	15–20	https://exrx.net/WeightExercises/Biceps/DBCurl
Triceps extension	seated triceps extension, kickback, triceps wall push-up	2	8–12	https://exrx.net/WeightExercises/Triceps/DBTriExt
Floor (chest) press	standing chest press, on ball, on bench, wall push-up, banded, chest fly	2	8–12	https://www.youtube.com/watch?v=uUGDRwge4F8
Glute Bridge	kickbacks, glute squeeze, seated leg extension, banded abduction, abduction, seated leg lifts, hip thrust variation	2	15–20	https://www.youtube.com/watch?v=wPM8icPu6H8
Bird dog	standing BD, seated diagonal raise, seated bird dog	2	10 (each side)	https://www.youtube.com/watch?v=wiFNA3sqjCA

**Instructions** **:**

1. Warm up (ex: https://www.youtube.com/watch?v=R0mMyV5OtcM, 7 August 2024). You may use any warm up that gets your blood flowing and gets you feeling mobile.

2. Perform each exercise (two sets for prescribed reps), with a **1–3-min rest** between each set. Then, move on to the next exercise.

3. Cool down (ex: https://www.youtube.com/watch?v=u5Hr3rNUZ24, 7 August 2024).

—Never perform an exercise that hurts, makes you tingle, or pinches. If you are noting anything uncomfortable, please bring it up to us at the next session. You may even film yourself doing the exercise and send it to the team, and we can comment on your form.

—Link to an article on exercise safety (not everything will be applicable, but please read the sections “when to stop exercising immediately” and “exercise in hot weather”) https://www.betterhealth.vic.gov.au/health/healthyliving/exercise-safety, 7 August 2024.

—What to bring: 1. Workout log 2. Equipment (dumbbells, soup cans, etc.) 3. Safe space 4. Towel 5. Water 6. Compression gear (if needed) 7. Proper shoes 8. A study chair.

**Table A3:** Missing data per variable in analysis for the resistance training intervention (*N* = 41).

	T1 *N* Missing	T2 *N* Missing	T3 *N* Missing
Total minutes of MVPA/week	1	4	6
Meet MVPA guidelines, % yes	1	4	3
Total minutes of RT/week	0	1	4
Meet RT guidelines, % yes	0	1	4
Meet MVPA and RT guidelines, % yes	1	5	4
5TSTS	3	8	-
Bicep curl volume	3	8	-
ESES	0	2	4
WAI	-	2	-
Match satisfaction	-	4	-
Match Quality	-	4	-
Match Similarity	-	4	-
Participant match communication	-	1	4

MVPA: moderate to vigorous physical activity; RT: resistance training; 5TSTS: Five times sit-to-stand; ESES: resistance training self-efficacy scale; WAI: Working Alliance Inventory (therapeutic alliance).

**Table A4:** Participant feedback on the challenges attending the qualified exercise professional (QEP)-led Zoom resistance training (RT) sessions and the partner sessions.

**Participant**	**Question 1: “Please comment on what made attending the (QEP-led zoom RT) session difficult or easy”:**
1	Zoom was fabulous!
2	Work, life, kid…
3	When I was away attending the sessions was not possible. Fortunately, I could use the the list of exercises sent us every week and was extremely grateful for the links to online demonstrations of each exercise. Great help for future home exercising!
4	Weekdays are busy until 5, so Tuesday was my only option. But I was ok with this, the program was flexible, which allowed me to spend some time with family AND learn some amazing stuff about exercising
5	Very flexible with several possible sessions. The QEP trainers were very proactive, optimistic, understanding of possible hurts and mentioned what we should watch out for, avoid or do more off. Felt they were qualified, and I loved being able to watch the different YouTube videos to propose different variations on a theme. Very helpful and motivating to practice with.
6	Traveling and work made it difficult
7	Timings were good so I could schedule my time and do it after work. Zoom video calls were so convenient. I probably would not have done this if it were in person due to timing, travelling, scheduling different. Thanks
8	Timing, unwell
9	Timing and having a young child in the home
10	There were several day/time options available, so I only missed 1 session because of travel.
11	The timing of them. Having kids and a day job made making the daytime/dinner time session almost impossible to make
12	The set time made it easy to remember, plus my husband working from home to remind me.
13	The sessions were at a good time for me, so they were easy to attend. the only one i missed was because i had covid.
14	The internet sometimes is not easy to connect.
15	The availability of session at different times and days were appreciated.
16	Post-surgery restrictions
17	No difficulties whatsoever
18	Look I tried no partner got covid
19	Kids home in the summer, other conflicts with zooms
20	It was great to do it over the lunch hour. I often did it in my office which was helpful. Personally, it was a good time of year to do something like this as during the week I was not as busy.
21	I was unable to attend the sessions available due to conflicting schedules
22	I was thankful for some different times to choose from- it made it better with my ever-changing schedule
23	I think the timing of the year was hard with summer vacations,, etc. I ended up doing the workouts by myself because I couldn’t make the times work (which is fine but not ideal)
24	I missed one session
25	I missed my final partner workout due to the national Rogers outage.
26	I had Covid so I missed a few sessions
27	I found this study harder because the meetings were not mandatory.
28	I did not have any difficulty attending the online sessions
29	Having a set time made it easy plus the sessions were always fun.
30	Frequency/timing of the sessions offered, accountability with my partner, disorganization on my part, I don’t feel like i set myself up for success from the beginning as I missed the intro session and watching it on YouTube wasn’t the same.
31	Fatigue made it hard. And life intruded at the end with the death of my brother.
32	Family schedule, travel
33	Exercises were very hard sometimes
34	Attending the session was made easy because it was immediately after 5pm, when I try to end my workday. If it was much later, I would have probably gotten lazy.
35	As I was in Greece and had major internet problems, I couldn’t commit to anything online.
36	After the 3rd session I could no longer do the workout, but I kept holding on hope that I could maybe later in the week with my triad so I attended all the way until the last week when I simply couldn’t anymore.
**Participant**	**Question 2: “What, if anything, made it difficult to communicate with or relate to your exercise partner?”**
1	We had different views on what support meant. I would have liked to work out with her, even if we didn’t connect via video. My partner wanted an email to confirm completion of a workout.
2	We had a 3-h time difference so that was a bit challenging
3	We both work with busy schedules
4	We both went on vacations at the same time; as we were in different time zones our communication was not possible.
5	Unfortunately, our schedules did not align well. I’m not sure about my partner, but the 8 weeks the sessions were running also ended up being busier than expected with commitments, etc.
6	Time zone difference
7	Time zone difference
8	Time difference. She was in BC, I’m in Ontario
9	Time difference
10	Sometimes we did not have the same time available to exercise together
11	She was unavailable
12	Scheduling difficulties of working out together at the same time.
13	Physical distance
14	Nothing
15	Not having a facilitated introduction and schedules that didn’t match.
16	No difficulties.
17	N/A
18	My partner’s work schedule, recreational activities, and home life meant that her schedule was quite limited (Friday evenings were free) and we could not line up our workouts. I think the summer means a lot of people are even more busy, so it was harder to connect.
19	Life got in the way – work was busy – busy with kid, etc.…
20	It would have been great to be able to meet by video, but she did not have the bandwidth. Both of us were quite busy but we were able to flex around out schedules.
21	It was around summer vacation which made it hard to schedule
22	I was physically unable to join past week 3
23	I was out of town for the last 4 weeks of the session and in a different time zone, so I wasn’t able to complete those weeks, unfortunately.
24	I was away too much at the beginning and I think I was too active for her so our goals didn’t seem to align.
25	Her work schedule.
26	Family’s – kid’s activities
27	Everything was EASY and gratifying

*Note.* Some spelling was corrected for publication. *n* = 5 and *n* = 14 participants left Question 1 and Question 2 blank, respectively.

## References

[1] Binkley J.M., Harris S.R., Levangie P.K., Pearl M., Guglielmino J., Kraus V., Rowden D. (2012). Patient perspectives on breast cancer treatment side effects and the prospective surveillance model for physical rehabilitation for women with breast cancer. Cancer.

[2] Mols F., Vingerhoets A.J., Coebergh J.W., van de Poll-Franse L.V. (2005). Quality of life among long-term breast cancer survivors: A systematic review. Eur. J. Cancer.

[3] Campbell K.L., Winters-Stone K., Wiskemann J., May A.M., Schwartz A.L., Courneya K.S., Zucker D.S., Matthews C.E., Ligibel J.A., Gerber L.H. (2019). Exercise guidelines for cancer survivors: Consensus statement from international multidisciplinary roundtable. Med. Sci. Sports Exerc..

[4] Gerland L., Baumann F.T., Niels T. (2021). Resistance exercise for breast cancer patients? Evidence from the last decade. Breast Care.

[5] Montaño-Rojas L.S., Romero-Pérez E.M., Medina-Pérez C., Reguera-García M.M., de Paz J.A. (2020). Resistance training in breast cancer survivors: A systematic review of exercise programs. Int. J. Environ. Res. Public Health.

[6] Schmitz K.H., Courneya K.S., Matthews C., Demark-Wahnefried W., Galvao D.A., Pinto B.M., Irwin M.L., Wolin K.Y., Segal R.J., Lucia A. (2010). American College of Sports Medicine roundtable on exercise guidelines for cancer survivors. Med. Sci. Sports Exerc..

[7] Brunet J., Taran S., Burke S., Sabiston C.M. (2013). A qualitative exploration of barriers and motivators to physical activity participation in women treated for breast cancer. Disabil. Rehabil..

[8] Smith-Turchyn J., Vani M., Sabiston C. (2020). Understanding how to reach the hard to reach in cancer rehabilitation. Glob. J. Nurs..

[9] Wurz A., St-Aubin A., Brunet J. (2015). Breast cancer survivors’ barriers and motives for participating in a group-based physical activity program offered in the community. Support. Care Cancer.

[10] Gonzalo-Encabo P., Wilson R.L., Kang D.W., Normann A.J., Dieli-Conwright C.M. (2022). Exercise oncology during and beyond the COVID-19 pandemic: Are virtually supervised exercise interventions a sustainable alternative?. Crit. Rev. Oncol. Hematol..

[11] Morrison K.S., Paterson C., Toohey K. (2020). The feasibility of exercise interventions delivered via telehealth for people affected by cancer: A rapid review of the literature. Sem. Oncol. Nurs..

[12] Winters-Stone K.M., Boisvert C., Li F., Lyons K.S., Beer T.M., Mitri Z., Meyers G., Eckstrom E., Campbell K.L. (2022). Delivering exercise medicine to cancer survivors: Has COVID-19 shifted the landscape for how and who can be reached with supervised group exercise?. Support. Care Cancer.

[13] Coughlin S.S., Caplan L., Stone R., Stewart J. (2019). A review of home-based physical activity interventions for breast cancer survivors. Curr. Cancer Rep..

[14] Sattar S., Haase K., Penz K., Effa C., Nedeljak J., Chalchal H., Souied O., Amir E., Pitters E., Campbell D. (2021). Feasibility of a remotely delivered strength and balance training program for older adults with cancer. Curr. Oncol..

[15] McDonough M.H., Beselt L.J., Daun J.T., Shank J., Culos-Reed S.N., Kronlund L.J., Bridel W. (2019). The role of social support in physical activity for cancer survivors: A systematic review. Psycho-Oncol..

[16] McDonough M.H., Beselt L.J., Kronlund L.J., Albinati N.K., Daun J.T., Trudeau M.S., Wong J.B., Culos-Reed S.N., Bridel W. (2021). Social support and physical activity for cancer survivors: A qualitative review and meta-study. J. Cancer Surviv..

[17] Smith-Turchyn J., Vani M.F., Murray R.M., McCowan M.E., Edward H., Nayiga B.K., Sabiston C.M. (2023). Peer support physical activity interventions partnering unknown survivors of cancer: A scoping review. Rehabil. Oncol..

[18] Krahn G.L. (1993). Conceptualizing social support in families of children with special health needs. Family Process.

[19] dos Santos W.D., Gentil P., de Moraes R.F., Ferreira Junior J.B., Campos M.H., de Lira C.A., Junior R.F., Bottaro M., Vieira C.A. (2017). Chronic effects of resistance training in breast cancer survivors. BioMed Res. Int..

[20] Yee J., Davis G.M., Hackett D., Beith J.M., Wilcken N., Currow D., Emery J., Phillips J., Martin A., Hui R. (2019). Physical activity for symptom management in women with metastatic breast cancer: A randomized feasibility trial on physical activity and breast metastases. J. Pain Symptom Manag..

[21] Tracey T.J., Kokotovic A.M. (1989). Factor structure of the working alliance inventory. Psychol. Assess. J. Consult. Clin. Psychol..

[22] Carr R.M., Prestwich A., Kwasnicka D., Thøgersen-Ntoumani C., Gucciardi D.F., Quested E., Hall L.H., Ntoumanis N. (2018). Dyadic interventions to promote physical activity and reduce sedentary behaviour: Systematic review and meta-analysis. Health Psychol. Rev..

[23] Pinto B.M., Stein K., Dunsiger S. (2015). Peers promoting physical activity among breast cancer survivors: A randomized controlled trial. Health Psychol..

[24] Ungar N., Sieverding M., Weidner G., Ulrich C.M., Wiskemann J. (2016). A self-regulation-based intervention to increase physical activity in cancer patients. Psychol. Health Med..

[25] Peck S.S., Vani M.F., Smith-Turchyn J., Sabiston C.M. (2023). Natural patterns of social support for physical activity participation in newly matched breast cancer survivor dyads. BMC Women Health.

[26] Dennis C.L. (2003). Peer support within a health care context: A concept analysis. Int. J. Nurs. Stud..

[27] Brunet J., Sabiston C.M., Burke S. (2013). Surviving breast cancer: Women’s experiences with their changed bodies. Body Image.

[28] Przezdziecki A., Sherman K.A., Baillie A., Taylor A., Foley E., Stalgis-Bilinski K. (2013). My changed body: Breast cancer, body image, distress and self-compassion. Psycho-Oncol..

[29] Sabiston C.M., Pila E., Vani M., Thogersen-Ntoumani C. (2019). Body image, physical activity, and sport: A scoping review. Psychol. Sport Exerc..

[30] Ginis K.A., McEwan D., Bassett-Gunter R.L. (2013). Physical activity and body image. Routledge Handbook of Physical Activity and Mental Health.

[31] Ginis K.A., Eng J.J., Arbour K.P., Hartman J.W., Phillips S.M. (2005). Mind over muscle?: Sex differences in the relationship between body image change and subjective and objective physical changes following a 12-week strength-training program. Body Image.

[32] Michie S., Van Stralen M.M., West R. (2011). The behaviour change wheel: A new method for characterising and designing behaviour change interventions. Implement. Sci..

[33] Howlett N., Schulz J., Trivedi D., Troop N., Chater A. (2019). A prospective study exploring the construct and predictive validity of the COM-B model for physical activity. J. Health Psychol..

[34] Willmott T.J., Pang B., Rundle-Thiele S. (2021). Capability, opportunity, and motivation: An across contexts empirical examination of the COM-B model. BMC Public Health.

[35] Schulz K.F., Altman D.G., Moher D. (2010). CONSORT 2010 statement: Updated guidelines for reporting parallel group randomised trials. J. Pharmacol. Pharmacother..

[36] Chan A.W., Tetzlaff J.M., Altman D.G., Laupacis A., Gøtzsche P.C., Krleža-Jerić K., Hróbjartsson A., Mann H., Dickersin K., Berlin J.A. (2013). SPIRIT 2013 statement: Defining standard protocol items for clinical trials. Ann. Intern. Med..

[37] Smith-Turchyn J., McCowan M.E., O’Loughlin E., Fong A.J., McDonough M.H., Santa Mina D., Arbour-Nicitopoulos K.P., Trinh L., Jones J.J., Bender J.L. (2021). Connecting breast cancer survivors for exercise: Protocol for a two-arm randomized controlled trial. BMC Sports Sci. Med. Rehabil..

[38] Segal R., Zwaal C., Green E., Tomasone J.R., Loblaw A., Petrella T. (2017). Exercise for people with cancer: A clinical practice guideline. Curr. Oncol..

[39] Von Elm E., Altman D.G., Egger M., Pocock S.J., Gøtzsche P.C., Vandenbroucke J.P. (2007). The Strengthening the Reporting of Observational Studies in Epidemiology (STROBE) statement: Guidelines for reporting observational studies. Lancet.

[40] Faul F., Erdfelder E., Buchner A., Lang A.G. (2009). Statistical power analyses using G* Power 3.1: Tests for correlation and regression analyses. Behav. Res. Methods.

[41] Sabiston C.M., Fong A.J., Smith-Turchyn J., Amireault S., Arbour-Nicitopoulos K.P., Bender J.L., Jones J.M. (2022). Exploring peer support characteristics for promoting physical activity among women living beyond a cancer diagnosis: A qualitative descriptive study. Oncol. Nurs. Forum.

[42] Cheema B., Gaul C.A., Lane K., Fiatarone Singh M.A. (2008). Progressive resistance training in breast cancer: A systematic review of clinical trials. Breast Cancer Res. Treat..

[43] Ratamess N.A., Alvar B.A., Evetoch T.E., Housh T.J., Ben Kibler W., Kraemer W.J. (2009). Progression models in resistance training for healthy adults. Med. Sci. Sports Exerc..

[44] Alleva J.M., Tylka T.L., Van Diest A.M. (2017). The Functionality Appreciation Scale (FAS): Development and psychometric evaluation in US community women and men. Body Image.

[45] Beesley H., Goodfellow S., Holcombe C., Salmon P. (2016). The intensity of breast cancer patients’ relationships with their surgeons after the first meeting: Evidence that relationships are not ‘built’ but arise from attachment processes. Eur. J. Surg. Oncol. (EJSO).

[46] Davison L.L. (2008). Embodying the Therapeutic Alliance: An Exploration of the Working Alliance in the Personal Trainer-Client Relationship. Master’s Thesis.

[47] Godin G., Shephard R.J. (1985). A simple method to assess exercise behavior in the community. Can. J. Appl. Sport. Sci..

[48] Short C.E., James E.L., Girgis A., D’Souza M.I., Plotnikoff R.C. (2015). Main outcomes of the Move More for Life Trial: A randomised controlled trial examining the effects of tailored-print and targeted-print materials for promoting physical activity among post-treatment breast cancer survivors. Psycho-Oncol..

[49] Amireault S., Godin G., Lacombe J., Sabiston C.M. (2015). The use of the Godin-Shephard Leisure-Time Physical Activity Questionnaire in oncology research: A systematic review. BMC Med. Res. Methodol..

[50] Santagnello S.B., Martins F.M., de Oliveira Junior G.N., de Freitas Rodrigues de Sousa J., Nomelini R.S., Murta E.F., Orsatti F.L. (2020). Improvements in muscle strength, power, and size and self-reported fatigue as mediators of the effect of resistance exercise on physical performance breast cancer survivor women: A randomized controlled trial. Support. Care Cancer.

[51] Bowman A., Denehy L., Benjemaa A., Crowe J., Bruns E., Hall T., Traill AEdbrooke L. (2023). Feasibility and safety of the 30-second sit-to-stand test delivered via telehealth: An observational study. PM R.

[52] Jones C.J., Rikli R.E. (2002). Measuring functional fitness of older adults. J. Act. Aging.

[53] Rikli R.E., Jones C.J. (2013). Senior Fitness Test Manual.

[54] Sagarra-Romero L., Butragueño J., Gomez-Bruton A., Lozano-Berges G., Vicente-Rodríguez G., Morales J.S. (2022). Effects of an online home-based exercise intervention on breast cancer survivors during COVID-19 lockdown: A feasibility study. Support. Care Cancer.

[55] Kroll T., Kehn M., Ho P.S., Groah S. (2007). The SCI exercise self-efficacy scale (ESES): Development and psychometric properties. Int. J. Behav. Nutr. Phys. Act..

[56] Megakli T., Vlachopoulos S.P., Thøgersen-Ntoumani C., Theodorakis Y. (2017). Impact of aerobic and resistance exercise combination on physical self-perceptions and self-esteem in women with obesity with one-year follow-up. Int. J. Sport Exerc. Psychol..

[57] Marsh H.W., Martin A.J., Jackson S. (2010). Introducing a short version of the physical self description questionnaire: New strategies, short-form evaluative criteria, and applications of factor analyses. J. Sport Exerc. Psychol..

[58] McDonald R.P. (1999). Test Theory: A Unified Treatment.

[59] Houghton C., Dowling M., Meskell P., Hunter A., Gardner H., Conway A., Treweek S., Sutcliffe K., Noyes J., Devane D. (2020). Factors that impact on recruitment to randomised trials in health care: A qualitative evidence synthesis. Cochrane Database Syst. Rev..

[60] Keilani M., Hasenoehrl T., Neubauer M., Crevenna R. (2016). Resistance exercise and secondary lymphedema in breast cancer survivors—A systematic review. Support. Care Cancer.

[61] Parkinson B., Meacock R., Sutton M., Fichera E., Mills N., Shorter G.W., Treweek S., Harmann N.L., Brown R.C.H., Gillies K. (2019). Designing and using incentives to support recruitment and retention in clinical trials: A scoping review and a checklist for design. Trials.

[62] Wrosch C., Scheier M.F., Carver C.S., Schulz R. (2003). The importance of goal disengagement in adaptive self-regulation: When giving up is beneficial. Self Identity.

[63] McEwan D., Rhodes R.E., Beauchamp M.R. (2022). What happens when the party is over?: Sustaining physical activity behaviors after intervention cessation. Behav. Med..

[64] Winters-Stone K.M., Dobek J., Nail L., Bennett J.A., Leo M.C., Naik A., Schwartz A. (2011). Strength training stops bone loss and builds muscle in postmenopausal breast cancer survivors: A randomized, controlled trial. Breast Cancer Res. Treat..

[65] Morishita S., Hamaue Y., Fukushima T., Tanaka T., Fu J.B., Nakano J. (2020). Effect of exercise on mortality and recurrence in patients with cancer: A systematic review and meta-analysis. Integr. Cancer Ther..

[66] Musanti R. (2012). A study of exercise modality and physical self-esteem in breast cancer survivors. Med. Sci. Sports Exerc..

[67] Cash T.F., Smolak L. (2011). Body Image: A Handbook of Science, Practice, and Prevention.

